# Immune repertoire profiling uncovers pervasive T cell clonal expansions in benign prostatic hyperplasia

**DOI:** 10.1172/JCI186939

**Published:** 2025-04-03

**Authors:** Anna S. Pollack, Christian A. Kunder, Chandler C. Ho, Josephine Chou, Andrew J. Pollack, Rachel L.P. Geisick, Bing M. Zhang, Robert B. West, James D. Brooks, Jonathan R. Pollack

**Affiliations:** 1 Department of Pathology, and; 2 Department of Urology, Stanford University School of Medicine, 300 Pasteur Drive, Stanford, California, USA.

**Keywords:** Immunology, Inflammation, Reproductive biology, Prostate cancer, T cells, Urology

## Abstract

We discovered T-cell clonal expansions in benign prostatic hyperplasia, indicative of a specific adaptive immune response and with implications for disease pathogenesis and new treatments.

**To the Editor:** Benign prostatic hyperplasia (BPH) is the nodular proliferation of stromal and epithelial elements within the prostatic transition zone (surrounding the proximal urethra) that occurs in older men (1). BPH leads to prostate enlargement and lower urinary tract symptoms that range from bothersome (increased hesitancy, urgency, and frequency) to potentially life-threatening (infection, acute urinary retention, and renal failure), and is often refractory to therapy (2). Chronic inflammation, in particular lymphocytic infiltrates, are thought to contribute to tissue remodeling and nodular proliferation that characterize BPH (3), but details remain unknown. Here, we applied immune repertoire analysis (4) — deep sequencing the collection of B cell receptor (BCR) and T cell receptor (TCR) gene rearrangements — of BPH nodules to investigate the lymphocytic response.

We microdissected 27 individual BPH nodules (comprising stromal, epithelial, and stromal-epithelial mixed nodules, from 17 patients) (Figure 1, A–D, Supplemental Figure 1, A–H, and Supplemental Table 1; supplemental material available online with this article; https://doi.org/10.1172/JCI186939DS1), each containing several thousand B and T lymphocytes. Immune repertoire analysis was done by PCR amplification and Illumina deep sequencing of BCR (immunoglobulin heavy chain framework region 2; *IGH* FR2) and TCR (TCR-β chain; *TRB*) variable-diversity-joining (VDJ) rearrangements. Across all the BPH nodules, B cell repertoires showed high diversity, with few individual BCR clonotypes — defined by shared V-J segments and complementarity-determining region 3 (CDR3) sequences — representing even 1% of the BCR repertoire (Figure 1E). In contrast, T cells exhibited marked clonal expansions, with several TCR clonotypes reaching 10%–30% of the TCR repertoire (Figure 1, F, and I–K, and Supplemental Table 2). Notably, T cell clonal expansions were significantly more prevalent among stroma-rich nodules (stromal and mixed) compared with epithelial nodules (Figure 1, F, and L–N). Lymphocytic infiltrates in epithelial nodules were often associated with glandular destruction (Supplemental Figure 1B), suggesting a distinct immune process. T cell clonal expansions were not merely a feature of aging, since they were significantly less apparent in non-BPH regions of the prostate (peripheral zone), or in blood of age-matched controls (Figure 1, G, H, and L–N). Among clonally expanded T cells, TCR CDR3 sequences — encoding epitope recognition sites — had predicted matches to known epitopes (including from Epstein-Barr virus and cytomegalovirus) in 15%–20% of clonotypes (Supplemental Table 2); thus, the vast majority of clonal TCRs had no predicted antigen pairing.

To characterize clonal T cell subsets, we performed 2-color RNA in situ hybridization (RISH) using clonotype-specific CDR3 probes to mark clonal T cells in 4 representative stroma-rich nodules. We identified coexpression of clonal *CDR3* sequences with *CD8A* but rarely *CD4*, revealing the clonal expansions to be of CD8^+^ (cytotoxic) T cells (Supplemental Figure 1, I–M). Classic cytotoxic T cells when activated release IFN-γ (*IFNG*) and TNF-α (*TNF*). Both IFN-γ and TNF-α stimulated proliferation of quiescent BPH stromal fibroblasts (Supplemental Figure 1, N–Q), providing a plausible link between T cell expansions and BPH nodular growth. While only 2%–5% of clonal T cells expressed *IFNG* and *TNF* (Supplemental Figure 1, R–V), up to 40% expressed the memory T cell marker IL-7R (*CD127*) (Supplemental Figure 1, W–Y), evidencing past episode(s) of antigen-driven T cell activation and cytokine release.

We previously reported that chemokine *CXCL13* — encoding a lymphocyte chemoattractant implicated in inflammatory conditions and autoimmune diseases — was the top differentially expressed gene in BPH versus normal prostate (5). Here, RISH revealed stromal CXCL13 expression in BPH stroma-rich nodules (Supplemental Figure 2, A–L). Such is reminiscent of tertiary lymphoid structures, although BPH stroma-rich nodules lack the dense lymphoid aggregates and organized B and T cell zones defining those structures. Notably, 5%–35% of clonally expanded T cells in BPH stroma-rich nodules expressed the CXCL13 receptor, *CXCR5* (Supplemental Figure 2, M–Q), which was less frequently expressed in CD8^+^ T cells from BPH epithelial nodules (Supplemental Figure 2, R–V). We therefore speculate that stromal CXCL13 expression may function in recruiting or retaining clonally expanded CD8^+^ T cells.

In summary, our immune repertoire analysis uncovered pervasive CD8^+^ T cell clonal expansions in BPH stroma-rich nodules. Our findings provide a plausible mechanistic connection between lymphocytic infiltrates and BPH nodular growth, mediated by antigen-driven T cell activation and release of proliferative cytokines. Possible shortcomings include reliance on prostates with coincident prostate cancer (albeit distant from the BPH), and that T cell subsets were characterized for only a subset of nodules. Further studies are needed to clarify functional subsets, spatiotemporal distributions, and clinical correlates of clonally expanded T cells, and to identify the inciting microbial and/or self-antigens. Nonetheless, our studies provide to our knowledge the first evidence of specific adaptive immune (T cell) responses in BPH. Our findings suggest potential new strategies for BPH prevention and treatment, including avoidance, immunization, or therapy against the inciting pathogens; targeting the CXCL13/CXCR5 axis to prevent lymphocyte recruitment; and blocking T cell cytokines, including IFN-γ and TNF, for which FDA-approved therapeutics are already used against hyperinflammatory and autoimmune disorders. Indeed, it is notable that TNF blockade (to treat autoimmune disease) was recently linked to decreased incidence of BPH (6).

Additional information is available in Supplemental Methods.

## Supplementary Material

Supplemental data

Supporting data values

## Figures and Tables

**Figure 1:**
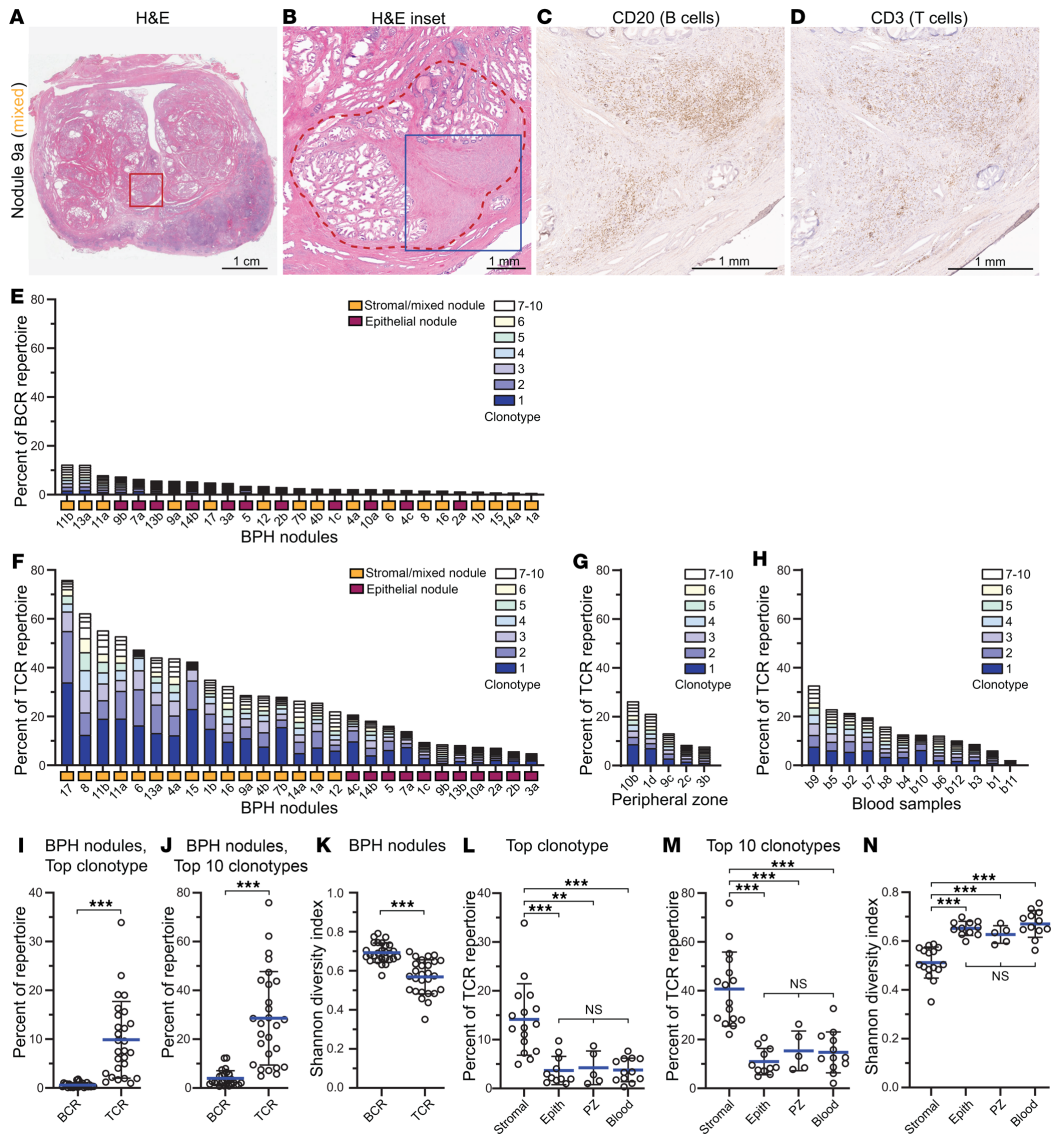
T cell clonal expansions are pervasive in BPH stroma-rich nodules. (**A**–**D**) Representative BPH mixed nodule (no. 9a) showing (**A**) H&E-stained transverse cross section through prostate (urethra center), with red square marking transition zone location of BPH nodule. (**B**) Magnified view with nodule border (and microdissected region) demarcated by red-dashed line. Blue square indicates further magnified region shown for immunohistochemistry (brown staining) of (**C**) CD20 (B cells) and (**D**) CD3 (T cells). Scale bars: 1 cm (**A**) and 1 mm (**B**–**D**). (**E**–**H**) Stacked bar graphs depict cumulative frequency of top 10 clonotypes for (**E**) BCR clonotypes across BPH nodules; and (**F**–**H**) TCR clonotypes across (**F**) BPH nodules (color-coded by nodule type), (**G**) normal prostate (peripheral zone, PZ), and (**H**) age-matched blood samples. Samples ordered by cumulative top 10 clonotype frequencies. (**I**–**N**) Scatter plots comparing (**I**–**J**) BCR versus TCR (**I**) top clonotype (frequencies), (**J**) top 10 clonotypes (cumulative frequencies), and (**K**) Shannon diversity index. Data are presented as mean ± 1 SD. ***P < 0.001 by 2-sided Student’s *t* test. NS, not significant. (**L**–**N**) Scatter plots comparing TCR (**L**) top clonotype (frequencies), (**M**) top 10 clonotypes (cumulative frequencies), and (**N**) Shannon diversity index among stroma-rich nodules, epithelial nodules, normal prostate (PZ), and blood. Data are presented as mean ± 1 SD. **P < 0.01, ***P < 0.001 by 1-way ANOVA with Tukey’s multiple-comparison test.

## Version 1 04/03/2025

In-Press Preview

## Version 2 06/02/2025

Electronic publication
