# Supplementary material for: Immune repertoire profiling uncovers pervasive T cell clonal expansions in benign prostatic hyperplasia
Source: J Clin Invest. 2025 Apr 3;135(11):e186939. doi: 10.1172/JCI186939 (PMC12126242; doi:10.1172/JCI186939)
Supplement: Supplemental data [file jci-135-186939-s127.pdf]

Immune repertoire profiling uncovers pervasive T-cell clonal expansions in benign prostatic hyperplasia

Anna S. Pollack[1], Christian A. Kunder[1], Chandler Ho[1], Josephine Chou[1], Andrew J. Pollack[1], Rachel L. P. Geisick[1], Bing Melody Zhang[1], Robert B. West[1], James D. Brooks[3], Jonathan R. Pollack[1,*]

Departments of [1]Pathology, and [2]Urology, Stanford University School of Medicine, 300 Pasteur Drive, Stanford, California, 94305

SUPPLEMENTAL TABLES, FIGURES, AND METHODS

Supplemental Table 1. Human BPH and blood specimens					
Patient ID	Samples[a]	Sex	Age (yrs)	Prostate size (cc)[b]	International Prostate Symptom Score (IPSS)[c]
1	1a (stromal), 1b (stromal), 1c (epithelial), 1d (PZ)	M	68	91	17
2	2a (epithelial), 2b (epithelial), 2c (PZ)	M	63	38	5
3	3a (epithelial), 3b (PZ)	M	69	19	17
4	4a (stromal), 4b (stromal-mixed), 4c (epithelial)	M	65	39	3
5	5 (epithelial)	M	69	60	16
6	6 (stromal-mixed)	M	57	41	2
7	7a (epithelial), 7b (stromal-mixed)	M	71	51	7
8	8 (stromal)	M	57	30	1
9	9a (stromal-mixed), 9b (epithelial), 9c (PZ)	M	61	107	na
10	10a (epithelial), 10b (PZ)	M	67	53	4
11	11a (stromal), 11b (stromal)	M	69	69	0
12	12 (stromal)	M	62	31	1
13	13a (stromal), 13b (epithelial)	M	66	45	1
14	14a (stromal), 14b (epithelial)	M	52	42	14
15	15 (stromal)	M	67	82	18
16	16 (stromal)	M	70	66	28
17	17 (stromal)	M	66	53	4
18	b1 (blood)	M	64	na	22
19	b2 (blood)	M	66	72	2
20	b3 (blood)	M	64	41	3
21	b4 (blood)	M	68	53	5
22	b5 (blood)	M	74	na	na
23	b6 (blood)	M	53	47	4
24	b7 (blood)	M	64	46	na
25	b8 (blood)	M	57	61	5
26	b9 (blood)	M	66	43	na
27	b10 (blood)	M	68	49	6
28	b11 (blood)	M	66	44	4
29	b12 (blood)	M	63	53	6

[a]PZ, peripheral zone

[b]Determined from pre-op prostate MRI

[c]Mild (0-7); Moderate (8-19); Severe (20-35)

na, not available

Supplemental Table 2. Top-10 TCR clonotype CDR3 sequences and their predicted antigen matches

Sample	Type	TRB V-gene	TRB J-gene	% Reads[a]	TRB CDR3 (nt)	TRB CDR3 (aa)	Predicted antigen match(es)[b]	CDR3 BaseScope
17	Stromal	Vb10-1	Jb2-2	33.894306	GCCAGCAGTTGGTTTCGCGGGGAGCTGTTT	ASSWFRGELF	0	X
17	Stromal	Vb7-8	Jb2-7	21.045582	GCCAGCAGCATGGGACAGGCCTACGAGCAGTAC	ASSMGQAYEQY	EBV, SARS-CoV-2, CMV	
11a	Stromal	Vb4-3	Jb1-6	19.107285	GCCAGCAGCCAGGGCTTACAGCCACCCCTCCAC	ASSQGLQPPLH	0	
11b	Stromal	Vb4-3	Jb1-6	19.002089	GCCAGCAGCCAGGGCTTACAGCCACCCCTCCAC	ASSQGLQPPLH	0	
7b	Mixed	Vb11-2	Jb1-2	15.623991	GCCAGCAGCTGGGGGGGAGGGTCCCACTATGGCTACACC	ASSWGGGSHYGYT	EBV, InfA, CMV	X
1b	Stromal	Vb14	Jb1-2	14.901462	GCCAGCAGCCAAGATCGAAGGGGGGGCTACACC	ASSQDRRGGYT	0	
6	Mixed	Vb2	Jb1-5	14.765678	GCCAGCAGCGGACTAGACAGGGTTGGGGTATATAGCAATCAGCCCCAGCAT	ASSGLDRVGVYSNQPQH	0	X
8	Stromal	Vb2	Jb1-1	12.437969	GCCAGCAAAGGGGACAGGGAAATGAACACTGAAGCTTTC	ASKGDREMNTEAF	0	
4a	Stromal	Vb28	Jb1-2	12.230172	GCCAGCAACACCCTAGGTACGGTTTATGGCTACACC	ASNTLGTVYGYT	0	
9a	Mixed	Vb4-3	Jb1-3	11.047346	GCCAGCAGCCCTGGGTCTAACTCTGGAAACACCATATAT	ASSPGSNSGNTIY	0	X
4c	Epithelial	Vb28	Jb1-2	9.7343972	GCCAGCAACACCCTAGGTACGGTTTATGGCTACACC	ASNTLGTVYGYT	0	
16	Stromal	Vb14	Jb1-2	9.6046035	GCCAGCAGCCAAGATCCTGGAGGGGTTTACGGCTACACC	ASSQDPGGVYGYT	0	
8	Stromal	Vb25-1	Jb1-2	9.1920875	GCCAGCAGTTCAGGGTTTGGCTACACC	ASSSGFGYT	SARS-CoV-2, InfA, CMV	
17	Stromal	Vb28	Jb2-7	8.0353452	GCCAGCAGTCCCCTTACGATTGAGAACGAGCAGTAC	ASSPLTIENEQY	0	
4b	Mixed	Vb28	Jb1-2	7.61271	GCCAGCAACACCCTAGGTACGGTTTATGGCTACACC	ASNTLGTVYGYT	0	
7a	Epithelial	Vb11-2	Jb1-2	7.4628112	GCCAGCAGCTGGGGGGGAGGGTCCCACTATGGCTACACC	ASSWGGGSHYGYT	EBV, InfA, CMV	
1a	Stromal	Vb7-8	Jb1-2	7.2506456	GCCAGCAGCTTAGCGTCAAGTGGGCGGGGCTACACC	ASSLASSGRGYT	0	
1b	Stromal	Vb7-8	Jb1-2	6.1998324	GCCAGCAGCTTAGCGTCAAGTGGGCGGGGCTACACC	ASSLASSGRGYT	0	
12	Stromal	Vb14	Jb1-1	5.9936986	GCCAGCAGCCAGTCGACACGGATTGGAGCTTTC	ASSQSTRIGAF	0	
13a	Stromal	Vb2	Jb2-4	5.1453433	GCCAGCAGTTCGCCTCTGGCAGTCAAAAACATTCAGTAC	ASSSPLAVKNIQY	0	
15	Stromal	Vb7-2	Jb2-1	4.5744907	GCCAGCAGCTTAGGCTATAGCGGGAGTAGCCCCTACAATGAGCAGTTC	ASSLGYSGSSPYNEQF	0	
13a	Stromal	Vb3-1	Jb2-1	3.961858	GCCAGCAGCCCCGGACCAAGCCAATTCTACAATGAGCAGTTC	ASSPGPSQFYNEQF	0	
16	Stromal	Vb19	Jb2-7	3.6173567	GCCAGTAGTACCCGGGGAGGGGCGGCCAACTACGAGCAGTAC	ASSTRGGAANYEQY	0	
4a	Stromal	Vb15	Jb1-1	3.4887263	GCCACCTATGCCTACCGGGACAGGGGCACTGAAGCTTTC	ATYAYRDRGTEAF	0	
9a	Mixed	Vb25-1	Jb1-4	3.354096	GCCAGCAGTGTACAGGGGAATGAAAAACTGTTT	ASSVQGNEKLF	0	
17	Stromal	Vb7-8	Jb2-7	3.288665	GCCAGCAGCATGGGACAGGCCTACGAGCAGTAC	ASSMGQAYEQY	EBV, SARS-CoV-2, CMV	
4a	Stromal	Vb4-1	Jb2-1	3.1079718	GCCAGCATCGGGGGGCCCGGAGAAAATGAGCAGTTC	ASIGGPGENEQF	0	
14a	Stromal	Vb11-2	Jb2-6	3.0449459	GCCAGCAGCCTCCCTCCTTCCGGGGGCAGGGGCTCTGGGGCCAACGTCCTGACT	ASSLPPSGGRGSGANVLT	0	
1c	Epithelial	Vb14	Jb1-2	2.9738664	GCCAGCAGCCAAGATCGAAGGGGGGGCTACACC	ASSQDRRGGYT	0	
17	Stromal	Vb6-4	Jb1-1	2.5522157	GCCAGCAGGCCACAGGGCTGGAACACTGAAGCTTTC	ASRPQGWNTEAF	0	
8	Stromal	Vb18	Jb2-2	2.5392783	GCCAGCTCACCGTTACCGGGAGGATACGAAATGAACACCGGGGAGCTGTTT	ASSPLPGGYEMNTGELF	0	
13a	Stromal	Vb4-1	Jb2-7	2.5022261	GCCAGCAGCCAACGGGATTCCGCAGGAGCCTCCTACGAGCAGTAC	ASSQRDSAGASYEQY	0	
9a	Mixed	Vb5-1	Jb2-7	2.2273157	GCCAGCAGCTTGGAAGGACAGGCGAGCTCCTACGAGCAGTAC	ASSLEGQASSYEQY	InfA, CMV, EBV, MART-1, SARS-CoV-2	
2a	Epithelial	Vb13	Jb1-1	2.1936274	GCCAGCAGCCACAGGAGGACGGGACAGGGGGAGAGGACTGAAGCTTTC	ASSHRRTGQGERTEAF	0	
16	Stromal	Vb4-2	Jb2-1	2.1192116	GCCAGCAGCCAAGAAGGGGGGACTATCGCGTACAATGAGCAGTTC	ASSQEGGTIAYNEQF	0	
2b	Epithelial	Vb13	Jb1-1	1.949884	GCCAGCAGCCACAGGAGGACGGGACAGGGGGAGAGGACTGAAGCTTTC	ASSHRRTGQGERTEAF	0	
13a	Stromal	Vb4-3	Jb2-2	1.8935765	GCCAGCAGCCCGACTAGCGGGACGGCCGGGGAGCTGTTT	ASSPTSGTAGELF	0	
12	Stromal	Vb25-1	Jb2-7	1.8227244	GCCAGCAGTGAATCGGGTTCCTACGAGCAGTAC	ASSESGSYEQY	SARS-CoV1, SARS-CoV-2	
9a	Mixed	Vb4-1	Jb1-2	1.779403	GCCAGCAGACCGACGGGGGGCTATAGTCACGGCTACACC	ASRPTGGYSHGYT	0	
13a	Stromal	Vb2	Jb2-1	1.7515583	GCCAGCAGTGAACCCGGGACTAGTAGCAATGAGCAGTTC	ASSEPGTSSNEQF	0	
9a	Mixed	Vb9	Jb2-2	1.702418	GCCAGCAGCGTAGGGGCTCCCGGGGAGCTGTTT	ASSVGAPGELF	0	
12	Stromal	Vb13	Jb2-7	1.6824201	GCCAGCACCCCGGGGCAGGGCAACTACGAGCAGTAC	ASTPGQGNYEQY	0	
14a	Stromal	Vb10-3	Jb2-3	1.6662339	GCCATCAGTGAGTCCGACGGGGATACGCAGTAT	AISESDGDTQY	0	
1a	Stromal	Vb14	Jb2-1	1.6253682	GCCAGCAGCCCCACGGGACTAGTCTTTGGGAATGAGCAGTTC	ASSPTGLVFGNEQF	0	
14b	Epithelial	Vb10-3	Jb2-3	1.6094015	GCCATCAGTGAGTCCGACGGGGATACGCAGTAT	AISESDGDTQY	0	
12	Stromal	Vb25-1	Jb1-1	1.5999606	GCCAGCAGTGATCCCCAGCAGGGTTTGCCTGAAGCTTTC	ASSDPQQGLPEAF	0	
12	Stromal	Vb2	Jb2-1	1.5864225	GCCAGCAGTTCGGTTCTCGGCAATGAGCAGTTC	ASSSVLGNEQF	0	
16	Stromal	Vb2	Jb1-5	1.5834825	GCCAGCAGCCAGGACAGGGACCGCTTATATAGCAATCAGCCCCAGCAT	ASSQDRDRLYSNQPQH	0	
13b	Epithelial	Vb3-1	Jb2-1	1.5605993	GCCAGCAGCCCCGGACCAAGCCAATTCTACAATGAGCAGTTC	ASSPGPSQFYNEQF	0	
1a	Stromal	Vb14	Jb1-2	1.5450071	GCCAGCAGCCAAGATCGAAGGGGGGGCTACACC	ASSQDRRGGYT	0	
1c	Epithelial	Vb7-8	Jb1-2	1.5196231	GCCAGCAGCTTAGCGTCAAGTGGGCGGGGCTACACC	ASSLASSGRGYT	0	
7b	Mixed	Vb2	Jb1-5	1.5100597	GCCAGCAGTGAAGGCGCAGTAACGAGCAATCAGCCCCAGCAT	ASSEGAVTSNQPQH	0	
11a	Stromal	Vb4-1	Jb2-2	1.5011213	GCCAGCAGCCTATCGGCATCTACCGGGGAGCTGTTT	ASSLSASTGELF	EBV, Yellow fever virus, SARS-CoV-2	
12	Stromal	Vb14	Jb1-2	1.4744252	GCCAGCAGCCAAACAGGGGGCGGCTACACC	ASSQTGGGYT	0	
14b	Epithelial	Vb11-2	Jb2-6	1.4321283	GCCAGCAGCCTCCCTCCTTCCGGGGGCAGGGGCTCTGGGGCCAACGTCCTGACT	ASSLPPSGGRGSGANVLT	0	
4b	Mixed	Vb4-1	Jb2-1	1.4007116	GCCAGCATCGGGGGGCCCGGAGAAAATGAGCAGTTC	ASIGGPGENEQF	0	
4a	Stromal	Vb5-1	Jb2-1	1.2773043	GCCAGCAGCTTAGGAGGGGACTCCTACAATGAGCAGTTC	ASSLGGDSYNEQF	0	
1b	Stromal	Vb14	Jb2-1	1.2740879	GCCAGCAGCCCCACGGGACTAGTCTTTGGGAATGAGCAGTTC	ASSPTGLVFGNEQF	0	
14a	Stromal	Vb9	Jb2-3	1.2609167	GCCAGCAGCGTCGTGGAGGGTTCGGGAGATACGCAGTAT	ASSVVEGSGDTQY	0	
17	Stromal	Vb10-1	Jb2-1	1.2573659	GCCAGCAACATCCTTAGGGGGAGGGGCTCCTACAATGAGCAGTTC	ASNILRGRGSYNEQF	0	
10a	Epithelial	Vb5-1	Jb2-7	1.2051173	GCCAGCAGCCCGGACGGGTACTACGAGCAGTAC	ASSPDGYYEQY	0	
1a	Stromal	Vb7-8	Jb1-2	1.1931032	GCCAGCAGCTTAGCGTCAAGTGGGCGGGGCTACACC	ASSLASSGRGYT	0	
10a	Epithelial	Vb2	Jb1-2	1.1902742	GCCAGCAGTCCTGGACAGGATCCACATGGCTACACC	ASSPGQDPHGYT	0	
17	Stromal	Vb14	Jb2-1	1.1647585	GCCAGCAGCCAATTCGCGAGGGGCCGAGACCGTGAGCAGTTC	ASSQFARGRDREQF	0	
1b	Stromal	Vb18	Jb1-2	1.119382	GCCAGCTCACCTCTTCTTTATGGCTACACC	ASSPLLYGYT	0	
4c	Epithelial	Vb2	Jb1-2	1.0427369	GCCAGCAGTTACATTACAGGGGGCGGCAATGGCTACACC	ASSYITGGGNGYT	0	
1b	Stromal	Vb12-4	none	1.0359004	GCCAGCAGAGCACAGGGGTCGGCTTTC	ASRAQGSAF	0	
1b	Stromal	Vb12-4	Jb2-7	1.0130423	GCCAGCAGTTTTGCCCCCCGGTACGAGCAGTAC	ASSFAPRYEQY	0	
13a	Stromal	Vb14	Jb1-2	1.0110346	GCCAGCAGCGCCCGGGACAGGGGGCGCATAGGCTATGGCTACACC	ASSARDRGRIGYGYT	0	
9a	Mixed	Vb14	Jb2-2	1.0025545	GCCAGCAGCCCCCTTGAAAGGGACACCGGGGAGCTGTTT	ASSPLERDTGELF	0	
9b	Epithelial	Vb4-3	Jb1-3	0.9833776	GCCAGCAGCCCTGGGTCTAACTCTGGAAACACCATATAT	ASSPGSNSGNTIY	0	
1b	Stromal	Vb7-8	Jb1-2	0.9736205	GCCAGCAGCTTAGCGTCAAGTGGGCGGGGCTACACC	ASSLASSGRGYT	0	
9b	Epithelial	Vb11-2	Jb2-2	0.9191228	GCCAGCAGGGGAGGAACTTCGAACACCGGGGAGCTGTTT	ASRGGTSNTGELF	0	
9b	Epithelial	Vb5-1	Jb1-2	0.9135354	GCCAGCAGCTTTAATTTAGGCAGGGGCCTCTATGGCTACACC	ASSFNLGRGLYGYT	0	
4c	Epithelial	Vb14	Jb2-7	0.9086603	GCCAGCAGCCTTTTACCGACAGCCGAGCAGTAC	ASSLLPTAEQY	0	
4b	Mixed	Vb5-1	Jb2-1	0.8996532	GCCAGCAGCTTAGGAGGGGACTCCTACAATGAGCAGTTC	ASSLGGDSYNEQF	0	
1a	Stromal	Vb12-4	Jb2-7	0.8807318	GCCAGCAGTTTTGCCCCCCGGTACGAGCAGTAC	ASSFAPRYEQY	0	
9b	Epithelial	Vb11-2	Jb1-2	0.8632491	GCCAGCAGCATCCAGACAGGGGGGAATGGCTACACC	ASSIQTGGNGYT	0	
9b	Epithelial	Vb14	Jb2-2	0.8436933	GCCAGCAGCCCAACAGGGACGAACACCGGGGAGCTGTTT	ASSPTGTNTGELF	0	
2b	Epithelial	Vb15	Jb1-2	0.8399847	GCCACCAGCAGAGAGGAAGGGGATGGCTACACC	ATSREEGDGYT	CMV	
6	Mixed	Vb4-3	Jb2-2	0.8149273	GCCAGCAGCCAAGGGATACCCCGAGCGGGGTACACCGGGGAGCTGTTT	ASSQGIPRAGYTGELF	0	
9b	Epithelial	Vb14	Jb1-1	0.8129627	GCCAGCAGCCAAGCTTGGGTAGGGGCTGAAGCTTTC	ASSQAWVGAEAF	0	
1c	Epithelial	Vb18	Jb1-2	0.7992228	GCCAGCTCACCTCTTCTTTATGGCTACACC	ASSPLLYGYT	0	
5	Epithelial	Vb27	Jb1-2	0.7850234	GCCAGCAGTATAAGTGGAGCTAACTATGGCTACACC	ASSISGANYGYT	0	
7b	Mixed	Vb19	Jb1-5	0.7676875	GCCAGTAGTACCGGGGACTCAAATCAGCCCCAGCAT	ASSTGDSNQPQH	CNV, EBV, SARS-CoV-2	
2a	Epithelial	Vb9	Jb1-1	0.7028538	GCCAGCACCTCCCGACAGGGAAAAGCTTTC	ASTSRQGKAF	0	
7b	Mixed	Vb30	Jb2-1	0.6860456	GCCTGGAGCCCCACTGTAAGCAATGAGCAGTTC	AWSPTVSNEQF	0	
1c	Epithelial	Vb12-4	none	0.6745734	GCCAGCAGAGCACAGGGGTCGGCTTTC	ASRAQGSAF	0	
10a	Epithelial	Vb15	Jb1-2	0.6728866	GCCACCAGTTATGGCAGGGGCGCCTATGGCTACACC	ATSYGRGAYGYT	0	
10a	Epithelial	Vb2	Jb1-2	0.6516822	GCCAGCAGTGGACAGGCCGTTAAAGATGGCTACACC	ASSGQAVKDGYT	0	
14b	Epithelial	Vb30	none	0.6383826	GCCTGGAGGGGAAACTTCACC	AWRGNFT	0	
9a	Mixed	Vb14	Jb2-7	0.6071316	GCCAGCAGCCAGGACTTTACCTACGAGCAGTAC	ASSQDFTYEQY	CMV	
7a	Epithelial	Vb14	Jb1-6	0.5994748	GCCAGCAGCCAGTTACTATCACCCCTCCAC	ASSQLLSPLH	0	
13b	Epithelial	Vb28	Jb1-5	0.5843586	GCCAGCAGAGTTCGGGGGAAGGGAAATCAGCCCCAGCAT	ASRVRGKGNQPQH	0	

10a	Epithelial	Vb7-9	Jb2-2	0.5817077	GCCAGCAGCTCGCCTACCGGGGAGCTGTTT	ASSSPTGELF	EBV	
7b	Mixed	Vb14	Jb2-7	0.5474441	GCCAGCAGCGAAACCGGGGGGAGGGGCGGCGAGCAGTAC	ASSETGGRGGEQY	0	
13b	Epithelial	Vb14	Jb1-2	0.5435536	GCCAGCAGCGCCCGGGACAGGGGGCGCATAGGCTATGGCTACACC	ASSARDRGRIGYGYT	0	
13b	Epithelial	Vb2	Jb2-4	0.5289254	GCCAGCAGTTCGCCTCTGGCAGTCAAAAACATTCAGTAC	ASSSPLAVKNIQY	0	
4c	Epithelial	Vb28	Jb2-6	0.5268335	GCCAGCAGTTTATTTCGGGGGAGTCTCCGCTCTGGGGCCAACGTCCTGACT	ASSLFRGSLRSGANVLT	0	
7b	Mixed	Vb12-5	Jb1-1	0.5221288	GCTAGTGCCCTGGGAATGGGGAACACTGAAGCTTTC	ASALGMGNTEAF	0	
7a	Epithelial	Vb14	Jb1-6	0.5215245	GCCAGCAGCCGACACGATTACTCCTATAATTCACCCCTCCAC	ASSRHDYSYNSPLH	0	
13b	Epithelial	Vb15	Jb2-2	0.5196865	GCCACCAGCCCGACTAGCGGGAATACGAACACCGGGGAGCTGTTT	ATSPTSGNTNTGELF	0	
2a	Epithelial	Vb18	Jb2-2	0.5169401	GCCAGCTCATATCGACGGGACACCGGGGAGCTGTTT	ASSYRRDTGELF	0	
14b	Epithelial	Vb4-3	Jb2-1	0.4969625	GCCAGCAGCCCTCTAGCGATTTCCTACAATGAGCAGTTC	ASSPLAISYNEQF	SARS-CoV-2	
15	Stromal	Vb14	Jb2-1	0.4873967	GCCAGCAGCCAAGATATGGGGCCACCTCAGAATGAGCAGTTC	ASSQDMGPPQNEQF	0	
1c	Epithelial	Vb18	Jb1-2	0.4772118	GCCAGCTCACGCCAGGAGGGGGGCTACACC	ASSRQEGGYT	0	
6	Mixed	Vb18	Jb1-5	0.4654113	GCCAGCTCACCACTCCCTACGGGACAGGGGGCGAGATTAGGTCAGCCCCAGCAT	ASSPLPTGQGARLGQPQH	0	
13b	Epithelial	Vb5-1	Jb1-6	0.4650232	GCCAGCAGCTCAGGGGGATACTCCTATAATTCACCCCTCCAC	ASSSGGYSYNSPLH	0	
4c	Epithelial	Vb4-1	Jb2-1	0.4583379	GCCAGCATCGGGGGGCCCGGAGAAAATGAGCAGTTC	ASIGGPGENEQF	0	
2a	Epithelial	Vb6-1	Jb1-2	0.4361081	GCCAGCAGCTTACAGGTTAACTATGGCTACACC	ASSLQVNYGYT	SARS-CoV-2	
10a	Epithelial	Vb10-3	Jb1-1	0.4353972	GCCATCAGTGATTCGACAGGGAATTCCGGTGAGGGAGCTTTC	AISDSTGNSGEGAF	0	
6	Mixed	Vb12-4	Jb1-5	0.4221031	GCCAGCGCCCGAGGTGGAAATCAGCCCCAGCAT	ASARGGNQPQH	0	
15	Stromal	Vb7-2	Jb2-1	0.4057213	GCCAGCAGCTTAGGCTATAGCGGGAGTAGCCCCTACAATGAGCAGTTC	ASSLGYSGSSPYNEQF	0	
3a	Epithelial	Vb11-2	Jb1-6	0.3921512	GCCAGCAGCCCTGGACAAGGGAATTCACCCCTCCAC	ASSPGQGNSPLH	CMV, SARS-CoV-2	
2b	Epithelial	Vb9	Jb1-1	0.3895908	GCCAGCACCTCCCGACAGGGAAAAGCTTTC	ASTSRQGKAF	0	
3a	Epithelial	Vb3-1	Jb1-2	0.3867247	GCCAGCAGCCAAGAATTTGGCTACACC	ASSQEFGYT	0	
7a	Epithelial	Vb6-4	Jb2-7	0.3749037	GCCAGCAGTGACTCACGCGGGAGACAGGGGAACTCCTACGAGCAGTAC	ASSDSRGRQGNSYEQY	0	
7a	Epithelial	Vb14	Jb1-2	0.36284	GCCAGCAGCCAAGTCTTAGGGACCAGCGGCTACACC	ASSQVLGTSGYT	0	
3a	Epithelial	Vb15	Jb1-1	0.3400573	GCCACGAGACAGGGAGAAGACTTGATCACTGAAGCTTTC	ATRQGEDLITEAF	0	
2b	Epithelial	Vb18	Jb2-7	0.3303961	GCCAGCTCACGGTCCGGACAGGGGGTCTACGAGCAGTAC	ASSRSGQGVYEQY	0	
2b	Epithelial	Vb6-1	Jb1-2	0.3178494	GCCAGCAGCTTACAGGTTAACTATGGCTACACC	ASSLQVNYGYT	SARS-CoV-2	
5	Epithelial	Vb12-4	Jb1-5	0.2106007	GCCAGCACCACGACGGGGCAGGTAGGAGCTCAGCCCCAGCAT	ASTTTGQVGAQPQH	0	
5	Epithelial	Vb7-8	Jb2-7	0.1891623	GCCAGCAGCATCGGACAGGCCTACGAGCAGTAC	ASSIGQAYEQY	EBV	

[a]Ranked by % reads

[b]Predicted matches from TCRmatch (IEDB database)

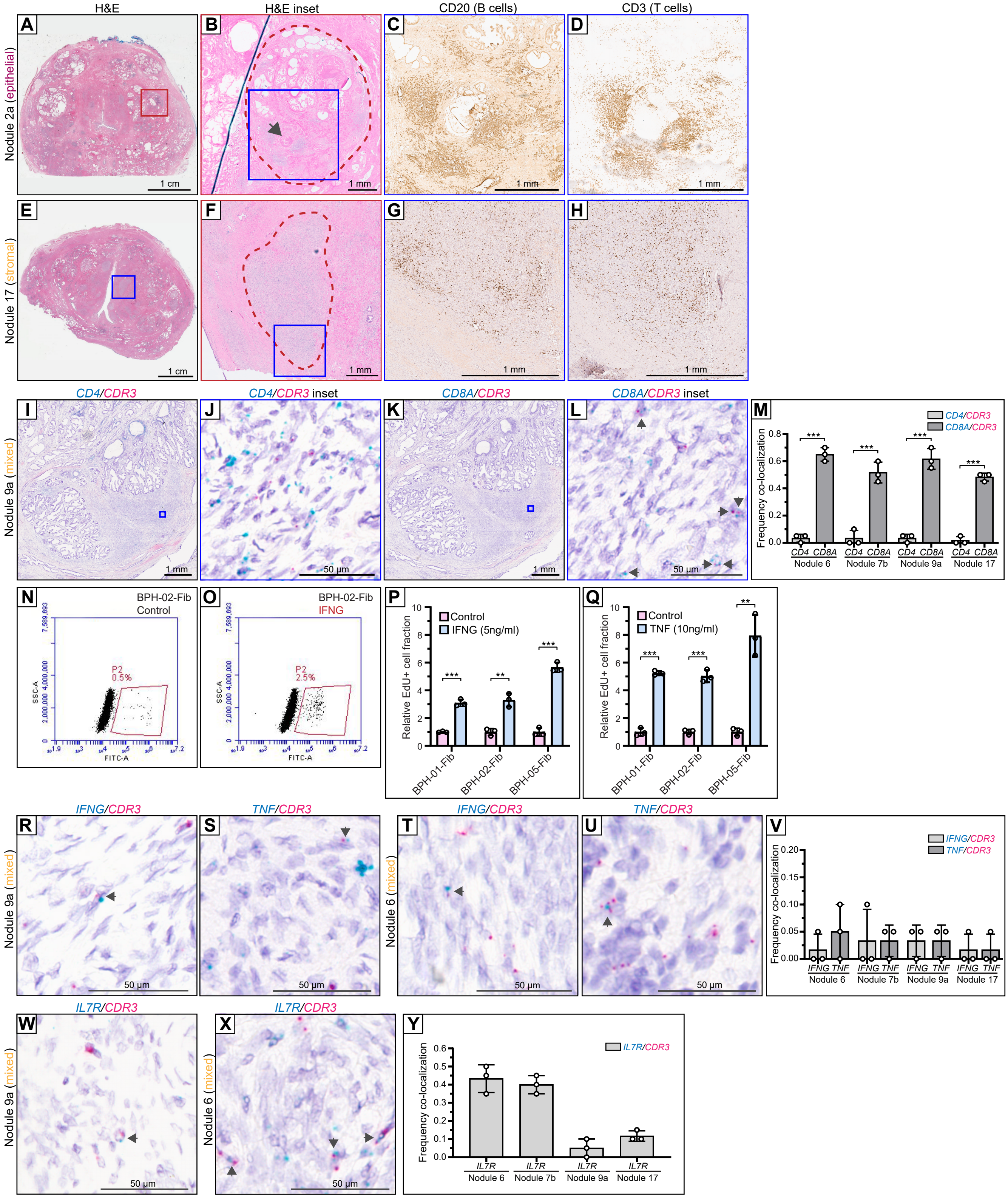

Supplemental Figure 1. Clonally-expanded T cells in BPH stromal-rich nodules are CD8+ (cytotoxic) T cells. (A-H) Representative BPH **(A-D)** epithelial nodule (#2a) and **(E-H)** stromal nodule (#17) showing **(A, E)** H&E-stained transverse section through prostate with red square marking transition zone location of selected nodule; **(B, F)** Magnified view with nodule border (and region microdissected for DNA) demarcated by dashed red line. Blue square indicates further-magnified region shown for IHC (brown-staining) of **(C, G)** CD20 (B cells) and **(D, H)** CD3 (T cells). **(I-L)** Two-color RISH of representative nodule (#9a, mixed) shows expression of **(I-J)** *CD4* (blue) and **(K-L)** *CD8A* (blue) versus TCR clonotype-specific *CDR3* sequence (red); arrows identify cells co-expressing *CD8A* and *CDR3*. **(M)** Bar graph quantifying co-localization frequency of *CD4*/*CDR3* and *CD8A*/*CDR3* in 4 different stromal/mixed nodules. Mean +/- 1 SD shown. ***, P-value < 0.001 (2-sided Student's t-test). **(N-O)** Representative flow cytometry dot plots of **(N)** control and **(O)** IFNG-induced cell proliferation of quiescent primary BPH fibroblasts (BPH-02-Fib), assayed by EdU incorporation (FITC channel). **(P, Q)** Bar graphs quantifying cell proliferation induced by **(P)** IFNG and **(Q)** TNF across 3 different quiescent primary BPH fibroblast samples. Mean +/- 1 SD shown. **, P-value < 0.01 ***, P-value < 0.001 (2-sided Student's t-test). Each experiment was conducted twice. **(R-U)** Two-color RISH of representative nodules (#9a and #6, mixed) shows expression of **(R, T)** *IFNG* (blue) and **(S, U)** *TNF* (blue) versus TCR clonotype-specific *CDR3* sequence (red); arrows identify cells co-expressing *IFN* or *TNF* together with *CDR3*. **(V)** Bar graph quantifying co-localization frequency of *IFNG*/*CDR3* and *TNF*/*CDR3* in 4 different stromal/mixed nodules. Mean +/- 1 SD shown. **(W-X)** Two-color RISH of representative nodules (#9a and #6, mixed) shows expression of *IL7R* (blue) versus TCR clonotype-specific *CDR3* sequence (red); arrows identify cells co-expressing *IL7R* and *CDR3*. **(Y)** Bar graph quantifying co-localization frequency of *IL7R*/*CDR3* in 4 different stromal/mixed nodules. Mean +/- 1 SD shown.

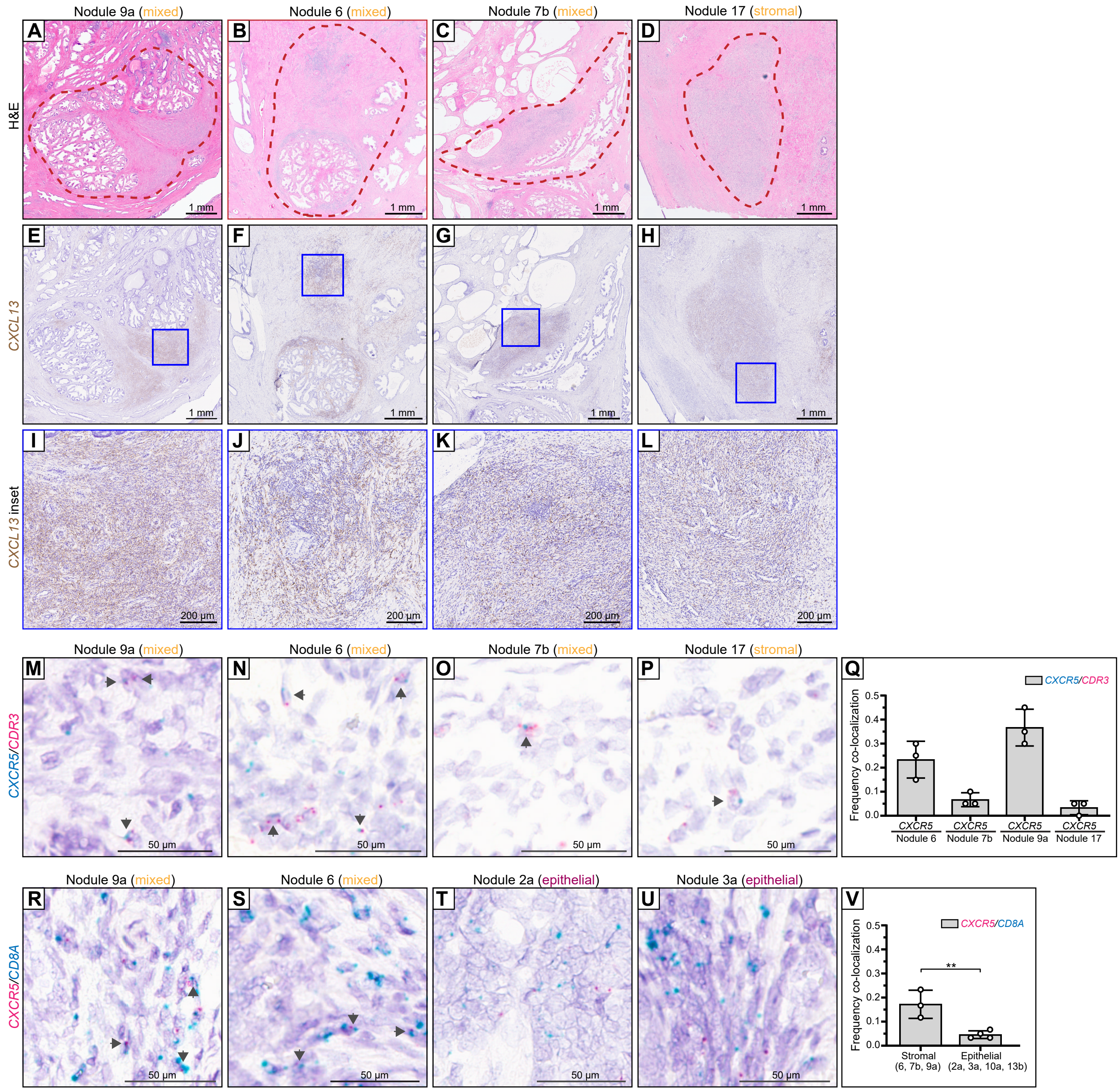

Supplemental Figure 2. Clonally-expanded T cells in BPH stromal-rich nodules express CXCL13 receptor, CXCR5. (A-L) RISH assays of *CXCL13* expression (brown staining) for four representative stromal/mixed nodules. **(A-D)** H&E stain with the nodule border (and region microdissected for DNA) outlined by a dashed red line. **(E-H)** *CXCL13* RISH assays and **(I-L)** corresponding insets with magnified view. Note, the H&E images shown here in panels A and D are reproduced from Figure 1B and Supplemental Figure 1F. **(M-P)** Two-color RISH of the above nodules shows expression of *CXCR5* (blue) versus TCR clonotype-specific *CDR3* sequence (red); arrows identify cells co-expressing *CXCR5* and *CDR3*. **(Q)** Bar graph quantifying co-localization frequency of *CXCR5*/*CDR3* in the 4 stromal/mixed nodules. Mean +/- 1 SD shown. **(R-U)** Two-color RISH of representative **(R-S)** stromal/mixed (9a and 6, mixed) and **(T-U)** epithelial (2a, 3a) nodules shows expression of *CXCR5* (blue) versus *CD8A* (red); arrows identify cells co-expressing *CXCR5* and *CD8A*. **(V)** Bar graph quantifying co-localization frequency of *CXCR5*/*CD8A* in 3 stromal/mixed versus 4 epithelial nodules. Mean +/- 1 SD shown. **, P-value < 0.01 (2-sided Student's t-test).

SUPPLEMENTAL METHODS

Sex as a biological variable

Our study examined male subjects only, because BPH is a disease exclusive to men.

Patient samples

All human specimens were obtained with Institutional Review Board approval and patient informed consent. Human prostate specimens were from radical prostatectomies done for prostate cancer treatment at the Stanford Hospital between 2017 and 2021. Study samples were selected from whole-mount cases where benign prostatic hyperplasia (BPH) transition zone (TZ) was distant from prostate cancer, and where BPH nodules – stromal, epithelial, or mixed (i.e., stromal nodules with substantial epithelial component) – contained microscopically-evident lymphocytes. The proportion of lymphocytes that were T cells ranged from approximately 40-60% (by immunohistochemistry). In a subset of representative cases, regions of lymphocyte-rich, normal prostate peripheral zone (PZ) were also sampled. Human blood specimens, matched for patient age and prostate size, were collected pre-operatively from patients undergoing radical prostatectomy done for prostate cancer treatment during 2023. Patient age, prostate size, and International Prostate Symptom Score (IPSS) are listed in Supplemental Table 1. Human primary BPH stromal fibroblast cultures (1, 2) were generated from surplus prostate tissue of cysto-prostatectomy cases (done for bladder cancer). Briefly, fresh BPH TZ tissue (with evident BPH nodules) was minced, then tissue disaggregated to single cells by sequential treatment with collagenase/hyaluronidase, trypsin, and dispase, following STEMCELL Technology's Prostate Tissue Dissociation protocol (3). Filtered single cells were then plated and passaged in a fibroblast-selective media, complete Stromal Cell Growth Media (Lonza), supplemented with

10nM metribolone (synthetic androgen) (Perkin Elmer). By the first passage, all cultured cells exhibited classic fibroblast spindle and stellate morphology.

BPH nodule microdissection and DNA isolation

BPH nodules were microdissected using the Pinpoint Slide DNA Isolation System (Zymo Research). Briefly, a dissecting microscope assisted the demarcation of individual BPH nodules on 10µM FFPE sections (guided by flanking H&E-stained slides). Pinpoint solution (a tissue adhesive) was applied by pipette tip to 10-15 serial 10µM sections, then the tissue detached and genomic DNA extracted using the QIAamp DNA FFPE Tissue Kit (Qiagen), and quantified by NanoDrop One. For blood samples, genomic DNA was isolated from buffy coats using the DNeasy Blood & Tissue Kit (Qiagen).

Immune Repertoire Profiling

Each sample containing 100 ng of genomic DNA underwent lymphocyte repertoire analysis using the LymphoTrack *IGH* (FR1/FR2/FR3) and *TRB* Assays (Invivoscribe) in a CLIA-certified molecular pathology laboratory (4). Barcoded gene-specific PCR primers were utilized to amplify the framework 2 of IGH and TRB repertoires flanking the BCR and TCR VDJ segments followed by deep-sequenced on a MiSeq (Illumina). Data processing, including de-multiplexing, read mapping (V(D)J gene assignment), CDR3 identification, and clonotype frequency (percent total reads) determination, was performed using LymphoTrack v2.4.3 software (Invivoscribe). BPH nodules were sequenced to an average read count of 90K (*IGH* FR2) and 130K (*TRB*). BPH nodule immune repertoires were estimated on average to represent approximately 3,800 different B-cell clonotypes and 1,900 different T-cell clonotypes, as determined by unique CDR3

sequences. Clonality/oligoclonality was operationally defined as one or more clonotype with ≥ 5% frequency. The Shannon equitability index (5) was calculated as described (4). TRB CDR3 epitope/antigen matches were predicted from TCRmatch (6) using the IEDB database (of infectious disease, allergy, autoimmunity, and transplantation antigens) (7) with default settings.

RNA in situ Hybridization and Immunohistochemistry

RNA in situ Hybridization (RISH) was done using manual BaseScope Duplex Assay (*CD4*, *CD*8A, *CXCR5*, *IL7R*, and *CDR3* regions), and RNAscope 2.5 HD Assay-Brown (*CXCL13*) according to the manufacturer (ACDBio). Chromogenic probes were Hs-CD4-C1, Hs-CD8A-C1, Hs-CXCR5-C1, Hs-CXCR5-C2, Hs-IL7R-C1, Hs-CXCL13-C1, and custom-designed TCR clonotype-specific CDR3(-C2) probes (Supplemental Table 2). Slides were scanned on an Aperio AT2 scanner. Co-localization was scored as red and blue signal spots either abutting or else clearly originating from the same cell. Co-localization was quantified by counting the number of co-localized signals per 20 *CDR3*+ cells, in each of three representative high-power (40X) fields. Immunohistochemistry was done using Tris-EDTA antigen retrieval, with ImmPRESS HRP Detection Kit (Vector Laboratories). Primary antibodies were CD20 (L26, 1:400) (Sigma), and CD3 (MRQ-39, 1:800) (Sigma).

Prostate cell culture experiments

Early-passage human primary BPH stromal fibroblasts were plated in 6-well plate wells (100K cells/well) in complete Stromal Cell Growth Media (SCGM; Lonza) (containing 5% FBS). The following day, the media was exchanged for low-serum SCGM (0-0.5% FBS) for 48 hrs to drive replicative quiescence (verified as < 1% EdU incorporation). Cells were then treated for 48 hrs

with physiological concentrations of recombinant human IFNG (PeproTech) (5ng/ml) or recombinant human TNF (10ng/ml) (PeproTech). DNA synthesis was then assayed by EdU incorporation using the Click-iT Plus EdU Flow Cytometry Assay Kit (Life Technologies). For each well, 10,000 cells were analyzed on an Accuri C6 flow cytometer (BD Biosciences). Each condition was assayed in biological triplicate, and each experiment was done twice.

Data availability

Raw data (Fastq files) for BCR and TCR repertoire analysis are available at the NCBI Sequence Read Archive (SRA) (accession PRJNA1218273). Supporting data values associated with the manuscript figures are available as supplemental material, in the Excel file "Supporting_data_values".

Statistics

Two-sided Student's t-test and one-way ANOVA (with Tukey's multiple comparisons test) were done to assess statistical significance, where $P < 0.05$ was considered significant. Graphs and charts were created using GraphPad Prism 10.0.

Study approval

The study was approved by the Stanford University Institutional Review Board (IRB #30642). Written informed consent was received from participants prior to including in the study.

AUTHOR CONTRIBUTIONS

ASP, CAK, BMZ, RBW, JDB and JRP conceived and planned the studies; ASP, JC, CH, RLPG, and JRP performed experiments; ASP, CH, AJP, BMZ and JRP analyzed data; ASP and JRP wrote the manuscript.

ACKNOWLEDGEMENTS

This study was supported by funding from the NIDDK (U54 DK130065) to J.D.B, R.B.W and J.R.P. We thank the Stanford University School of Medicine core facilities providing services, including the Stanford Tissue Bank and the Human Pathology/Histology Service Center.

REFERENCES

1. Peehl DM, Sellers RG. Cultured stromal cells: an in vitro model of prostatic mesenchymal biology. *Prostate*. 2000;45(2):115-123.
2. Zhao H, Ramos CF, Brooks JD, Peehl DM. Distinctive gene expression of prostatic stromal cells cultured from diseased versus normal tissues. *J Cell Physiol*. 2007;210(1):111-121.
3. STEMCELLTechnologies. A guide to solid mammary and prostate tissue dissociation. *Technical Bulletin*. 2009;29182.
4. Ho CC, Tung JK, Zehnder JL, Zhang BM. Validation of a Next-Generation Sequencing-Based T-Cell Receptor Gamma Gene Rearrangement Diagnostic Assay: Transitioning from Capillary Electrophoresis to Next-Generation Sequencing. *J Mol Diagn*. 2021;23(7):805-815.
5. Shannon CE. A mathematical theory of communication. *Bell Syst Tech J*. 1948;27:379-423.
6. Chronister WD, et al. TCRMatch: Predicting T-Cell Receptor Specificity Based on Sequence Similarity to Previously Characterized Receptors. *Front Immunol*. 2021;12:640725.
7. Vita R, et al. The Immune Epitope Database (IEDB): 2018 update. *Nucleic Acids Res*. 2019;47(D1):D339-D343.
